# Predicting and preventing COVID-19 outbreaks in indoor environments: an agent-based modeling study

**DOI:** 10.1038/s41598-022-18284-8

**Published:** 2022-09-27

**Authors:** Mardochee Reveil, Yao-Hsuan Chen

**Affiliations:** 1 Corning Incorporated, Corning, NY USA; 2 Merck & Co., Inc., Kenilworth, NJ USA

**Keywords:** Infectious diseases, Computational models, Computational platforms and environments, Health policy, Occupational health, Epidemiology

## Abstract

How to mitigate the spread of infectious diseases like COVID-19 in indoor environments remains an important research question. In this study, we propose an agent-based modeling framework to evaluate facility usage policies that aim to lower the probability of outbreaks. The proposed framework is individual-based, spatially-resolved with time resolution of up to 1 s, and takes into detailed account specific floor layouts, occupant schedules and movement. It enables decision makers to compute realistic contact networks and generate risk profiles of their facilities without relying on wearable devices, smartphone tagging or surveillance cameras. Our demonstrative modeling results indicate that not all facility occupants present the same risk of starting an outbreak, where the driver of outbreaks varies with facility layouts as well as individual occupant schedules. Therefore, generic mitigation strategies applied across all facilities should be considered inferior to tailored policies that take into account individual characteristics of the facilities of interest. The proposed modeling framework, implemented in Python and now available to the public in an open-source platform, enables such strategy evaluation.

## Introduction

The coronavirus disease 2019 (COVID-19) pandemic, caused by severe acute respiratory syndrome coronavirus 2 (SARS-CoV-2) with first onset of cases dating back to November 2019, has infected more than 500 million people causing more than 6 million fatalities globally (with more than 992,000 in the U.S. alone) as of May, 2022^1–3^. Its social, economic, and health impact on people’s livelihood has been broad and profound, with some racial minority groups in the U.S. bearing a disproportionate burden^4,5^.

As SARS-CoV-2 continues its global spread, the emerging global data reveal that age-specific mortality rates range from 0.001% (95% credible interval, 0–0.001%) in those aged 5–9 years old to 8.29% (95% credible intervals, 7.11–9.59%) in those aged 80+^6^, as compared to 0.06% for seasonal influenza which caused an estimated 22,000 deaths during the 2019-2020 season in the U.S.^7^. The relatively higher fatality rate of COVID-19 and the resulting significant pressure on healthcare resources have left key hospital services in crisis, risking long delays in admitting new COVID-19 patients and extremely sick non-COVID-19 patients.

To fully contain the spread of the virus, policymakers around the world cannot rely on vaccination alone ; instead they have to develop strategies consisting of a mixture of vaccinations, pharmaceutical interventions (i.e., treatments), as well as non-pharmaceutical interventions, such as school closures, cancellation of mass gatherings, physical distancing, and mask wearing.

Companies and institutions with in-person operations also have a responsibility to develop customized closure/reopening plans for their facilities to protect their workforce and facility occupants. The decision makers in these institutions need to pay close attention to indoor transmission risks, especially due to the higher probability of close contact compared to outdoor environments^8–10^. In doing so, they must take into account the main factors of interest for indoor transmission including environmental factors (proximity, duration, shared air, shared surfaces, etc.), human factors (mask wearing, social distancing, hygiene behaviour, age, physical distancing, contact frequency, etc.) as well as epidemiological factors (virus half-life, viral load through different expiratory events, etc.)^11,12^.

To understand the risks associated with the pandemic, several companies, governments and health agencies have relied on smartphone tagging, wearable devices, CCTV and other tracking methods to identify close contacts^13–16^. Those methods offer contact tracing and occupancy management features including proximity detection, motion analysis, people counting, etc. However, they raise serious privacy concerns and are useful only for real-time or historical analysis^13,16,17^. They cannot be used for what-if analysis for different facility occupancy scenarios and for future predictions of infections based on current facility usage.

In this study, we develop an agent-based simulation framework for indoor environments that takes into account individual schedules, occupancy level, facility layout, as well as pedestrian traffic across facilities. This new framework enables decision makers to compute realistic contact networks and generate risk profiles of their facilities without relying on wearable devices, smartphone tagging or surveillance cameras. Here we present the overall framework, demonstrate its use in model facilities, and discuss lessons learned from analysis of current results. We hope this study, together with the open-source release of the code, can provide sufficient information for others to use this framework for their needs.

## Background and related work

Agent-based modeling (ABM) is a class of computational models for simulating the actions and interactions of autonomous agents. ABM is a powerful simulation modeling technique that has been widely applied to various real-world business problems, including in the field of infectious disease modeling^18–23^.

Several ABM studies have been performed since the start of the pandemic to help in the fight to control the disease spread. The list includes those especially built for the purpose of forecasting^24–27^. Various ABMs have also been proposed for what-if scenario analysis at the scale of countries, cities or small towns^28–33^. Most models, however, are not designed to be used at the scale of a single facility, and those that do, do not include specific facility layouts nor realistic occupant scheduling and circulation^34^. Table 1 shows a high-level overview of some of the tools developed by various research teams. They can be divided in different categories based on the simulation scale (country/city or facility/building), whether the simulation includes spatial information or not and if so whether it is a simple (typically rectangular) grid or real maps.

**Table 1:** Examples of recent COVID-19 agent-based modeling publications.

Name	Scale	Spatial domain
Covasim^33^	Country/City	None
CoPE^35^	Country/City	None
PanSim^36^	Country/City	Real map
CityCOVID^37^	Country/City	Real map
N/A^34^	Facility/Building	Rectangular grid
OccSim^38^	Facility/Building	Real map
This Work	Facility/Building	Real map

Representative tools that work at the scale of a country or city includes Covasim^33^, CoPE^35^, PanSim^36^, and CityCOVID^37^. Covasim takes demographic information and the contact network of the population of interest as well as possible pharmaceutical and non-pharmaceutical interventions as inputs and outputs the daily number of infections. Unlike in Covasim, CoPE includes individual choices in its formulation. This allows some agents to be “non-compliers” with respect to various policy interventions. Both Covasim and CoPE do not support spatial information in their formulation. PanSim and CityCOVID are two examples of country or city-wide ABM simulation tools with support for real maps. Both tools generate virtual equivalents of real cities to model the spread of the disease while the local population go through their regular daily activities. The advantage of incorporating real maps are the followings: (1) generate more realistic contact networks (2) offer detailed insights into the locations where most infections occur and (3) allow the evaluation of more nuanced intervention strategies (e.g. close only schools in district 1).

Compared to country or city-wide, ABM simulations at the scale of a single facility offer the additional benefit of providing a more granular level of insights to control the spread of the pandemic in localized environments. While there is a long list of studies and tools for modeling at the scale of a country or city, there is only a limited set at the scale of a facility. Erik Cuevas published one of the first examples of a COVID-19 ABM simulation at the scale of a single facility. While the study emulates individual movements within facilities, it does not explicitly encode spatial information in a real map. OccSim, another facility-scale example, takes real facility maps and realistic individual behaviors into account; however, the code is not made available to the community.

Results from country or city-wide simulations help government officials make sound policy decisions. However, they are usually too general and high-level to be useful in the local context of a single facility. This is because typical assumptions such as indistinguishable agents while valid at large scales, fail in the local context. Moreover, large scale simulations typically care about large scale interventions such as city-wide school closures, no indoor dining, etc. while facility administrators care more about interventions such as how many entrances to keep open and at which capacity to run their facility. The current work is intended to fill this gap by helping facility administrators develop a good understanding of the risk profiles of their own facilities and the trade-off afforded by various policy decisions.

## Methods

**Figure 1:**
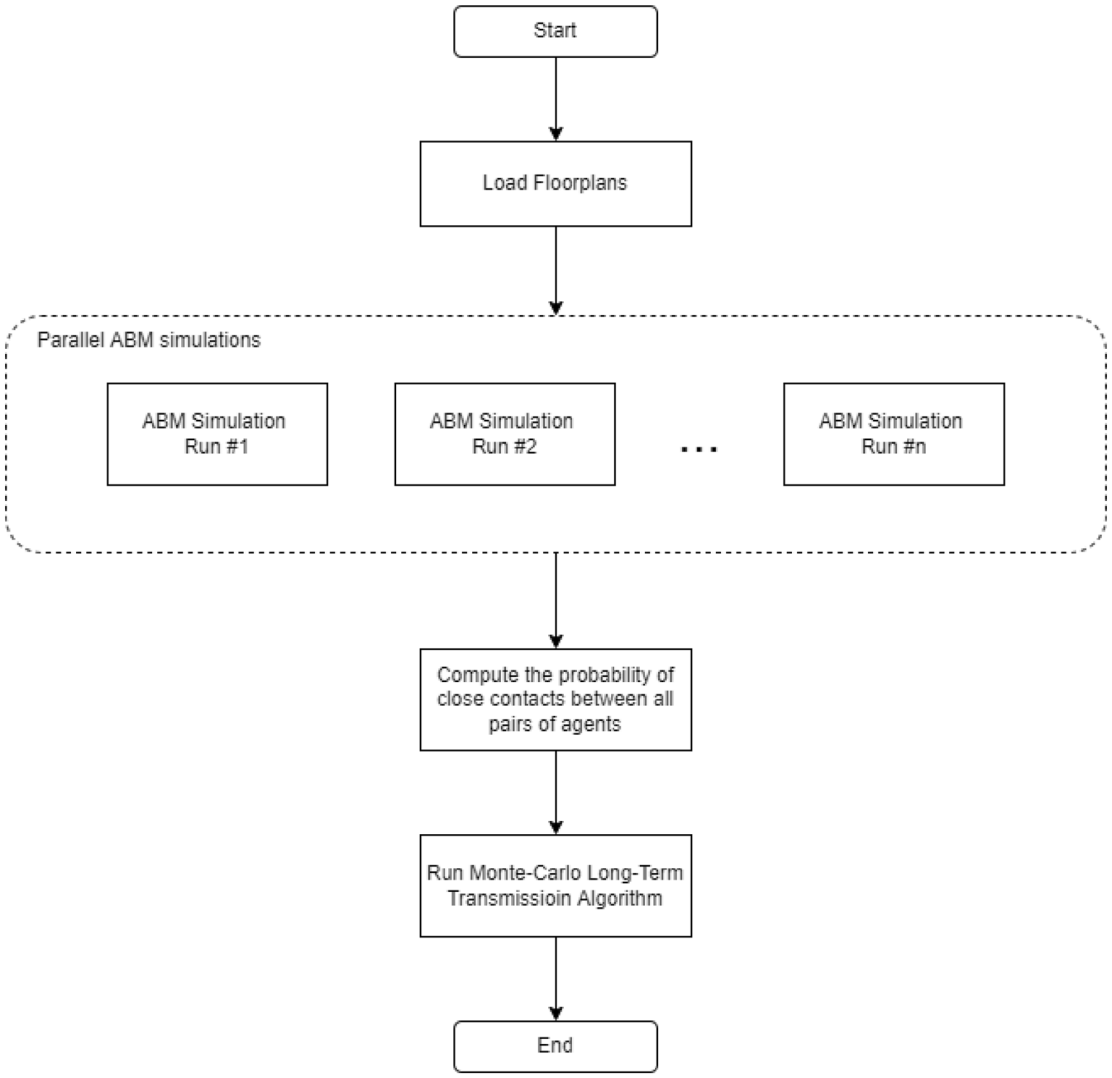
Flowchart describing the long-term transmission framework used in this work. Parallel 1-day ABM simulations are performed to estimate the probability that any pair of agents will make contact resulting in a successful transmission. This probability is then used as an input to a Metropolis Monte Carlo long-term transmission algorithm to study the dynamics of transmission over long-time horizons.

We propose a new simulation framework that sequentially combines agent-based modeling with Monte Carlo simulations for long-term transmission studies. The overall framework is shown in Fig. 1. Parallel ABM simulations are used to compute the daily probability of close contact ($p_{cc}$) between agents. We assume daily schedules are independent (from 1 day to the next) for a given agent and break multi-day simulations down into multiple 1-day simulations that can be performed in parallel.

Outputs from this set of ABM simulations are used to generate a contact network represented by a probability matrix assumed to be a function of agent scheduling and facility layout, and NOT a function of whether agents are infected or not. In other words, this probability matrix would be valid whether there is a pandemic or not since it is computed based on how the facility is used during normal operation.

The estimated probabilities of having transmissible contacts among any pair of two agents ($p_{cc}$) based on the parallel run results are then used in a Monte Carlo Long-Term Transmission Algorithm (MC-LTTA) to study the disease progression. Within the Monte Carlo simulations, $p_{cc}$ is used to update the infection status of each facility daily to predict indoor transmission profiles over long-time horizons. The proposed framework, in one of the best-case scenarios, has led to a speed-up on the order of the number of days of interest compared to an equivalent multi-day simulation.

In the following sections, we describe each step of this workflow in more details. We start by explaining key elements of the facility-aware agent-based simulations such as how the facility floorplans are managed, how individual schedules are generated, and how indoor navigation is implemented. This is followed by how the probability of close contact is defined and computed. Finally, we explain the Monte Carlo simulations and how they are used to compute long-term transmission profiles.

### Facility-aware agent-based models

#### Facility floor plans

In our ABM simulations, we divide the simulation domain into spaces defined by their physical boundaries (i.e., walls), doorways for circulation between spaces, and functionalities (i.e., restroom, meeting rooms, office space, etc.). The details of the simulation domain are automatically extracted from user-provided floorplan data. A key challenge addressed in the open-source implementation of this framework is how to incorporate user-provided floorplans. Floorplans of corporate facilities are managed in various CAD (Computer-Assisted Design) formats and with different levels of details based on the type of facility and the skillset of the people who manage them. This makes it harder to use this framework in practice. To facilitate the use of arbitrary floorplans, a simplified SVG format is used to represent the boundaries of each space and all valid doorways. Each space is assigned a unique identifier and a keyword string corresponding to its expected usage. Floorplans represented in this simplified format can be automatically processed by the platform.

#### Agent schedules

In this demonstrative study, personnel schedules are generated automatically based on a predefined list of activities such as cafeteria visit, restroom visit, office work, meetings, etc. Each activity is defined by a minimum and maximum duration as well as a minimum and maximum number of occurrences during the day. The type of activity determines where agents must be physically present for the activity to occur. In case multiple spaces are available for a given type of activity, the location is chosen randomly from the list of supported spaces. In cases where part of (or the full) the daily schedule of agents is known in advance (e.g., lunch occurs at given timeframe and place), these can be pre-added to agent’s schedules. Agents are assigned a primary office (or workstation) to which they will go at the beginning of their day and come back to periodically. The scheduling algorithm also supports policies around meetings such as maximum attendance, maximum room occupancy, etc. The scheduling routine described here was designed with a research facility in mind, but custom scheduling routines can be designed and added to the open-source code to match the typical usage of any facility of interest. Schedules are generated separately for each day; however, the same office assignment is kept for runs belonging to the same simulation set.

#### Indoor navigation

During the ABM simulations, for agents to follow their daily schedules, they must move from space to space according to the location set for each activity. To be realistic, their motion within the facility cannot be random (in real life, people go from space to space in a well-defined manner). To address this requirement, we develop an indoor navigation algorithm that computes a suitable route that agents can follow to move within the facility. At ingestion time, a navigation network is computed for each facility. This network is then used in combination with each agent’s schedule to compute a suitable itinerary for them at simulation time. In the default setting, agents pick the best route (typically the shortest path) between a starting point and a destination within the facility. Specific doors labeled as entrances/exits for the facility are used to define where each agent will enter and exit the facility at the beginning and end off their workday respectively. Contacts with other agents is tracked both during transit and when they are stationary (e.g., during meetings).

### Computing the probability of close contact

According to U.S. Center for Disease Controls (CDC)’s definition as of December 2020, a close contact/transmissible event is defined to be a situation when “someone who was within 6 feet of an infected person for at least 15 min over a 24-h period, starting from 2 days before illness onset (or, for asymptomatic clients, 2 days prior to positive specimen collection) until the time the patient is isolated” This definition of transmissible contact is adopted in our model.

As agents follow their schedules and associated itineraries within the facility, they naturally encounter one another. The model can keep track of the transmissible contacts among all pairs of agents that are not separated by physical walls, with detailed notes on location, duration, as well as the specific agents involved. Through the ABM simulations, we can obtain information on $D_{ij}$, the cumulative contact duration (in the unit of minutes) between any pair of agents i and j during a specific time period (e.g., 1 day).

In the example to showcase the utility of this model, we assume that the probability of infection during a close contact with an infectious individual is 100%. As a result, the transmission probability function, $f(D_{ij})$, can be defined as a delta function (Eq. 1), such that it is equal to 1 when the recorded cumulative contact duration is 15 min or more, and 0 otherwise. A more realistic expression for $f(D_{ij})$ could take a different form, such as a parameterized sigmoid (with parameters that account for personal protective equipment, stage of infection, etc.^39–43^. The current definition is chosen only for demonstrative purposes. As newer and more epidemiologic evidence emerges, the function $f(D_{ij})$ can be updated accordingly.

1 $$\begin{aligned} f^{CDC}(D_{ij}) = {\left\{ \begin{array}{ll} 1 &{} \text {if }D_{ij} >= 15\\ 0 &{} \text {otherwise} \end{array}\right. } \end{aligned}$$

Moreover, the probability that any two agents i and j have a cumulative contact duration of 15 min or more in any given day, $P(D_{ij} \ge 15)$, is estimated by taking the average of $f_k^{CDC} (D_{ij})$, the transmission probability function as defined earlier between agents i and j for day k, over a total of N days (Eq. 2).

2 $$\begin{aligned} P(D_{ij} \ge 15) = \frac{\sum _{k=1}^{k \le N} f_{k}^{CDC}(D_{ij})}{N} \end{aligned}$$

### Long-term transmission model

**Figure 2:**
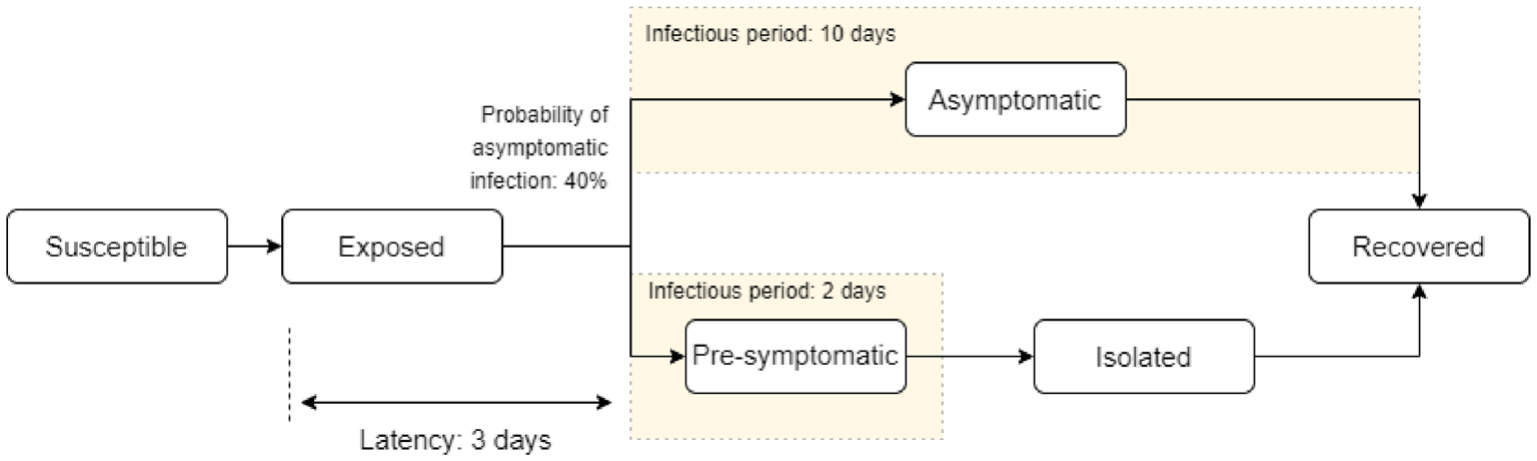
The different states in the Long-Term Transmission model. Susceptible individuals are exposed according to a probability of close contacts computed from ABM simulations. We assume symptomatic individuals are not allowed in the facility and no deaths occur within the population of facility occupants.

To increase the computational speed for epidemiologic studies that take into consideration detailed floor layouts of large facilities as well as complex personnel scheduling, we propose the Monte Carlo Long-Term Transmission Algorithm (MC-LTTA), a heuristic long-term transmission modeling framework summarized in Fig. 2 for long-term indoor transmission studies. A pseudo-code description of the MC-LTTA is provided in the supplemental information.

In the MC-LTTA, for each day, we use the probability of close contact between infectious and susceptible agents as computed from ABM simulations to determine who gets infected. We assume a 40% probability of asymptomatic infections which falls well within the range of 30% to 45% as reported in the literature^44–46^. The latency, pre-symptomatic and asymptomatic periods were chosen to be 3, 2 and 10 days, respectively, based on estimates from the literature^44,47–50^. We include a sensitivity analysis around these parameter values in the supplemental material.

Each day, the non-susceptible population is updated by adding all agents that have reached the end of their infection period. We note that recent data showing that reinfections are possible may invalidate this assumption. Currently infected individuals are also assumed to be non-susceptible. We only consider asymptomatic and pre-symptomatic agents as infectious because symptomatic individuals are not allowed in most facilities. In case such policy does not exist for the facility of interest, a suitable percentage of the full list of infected agents should be used instead. Potential infections that occur outside of the facility can be taken into account by updating the list of daily infected agents with a number of randomly selected susceptible agents corresponding to the rate of infection in the surrounding community. Similarly, if the facility enforces a regular testing policy, the list of asymptomatic agents can be updated accordingly as they are tested and found to be infected.

## Results

**Figure 3:**
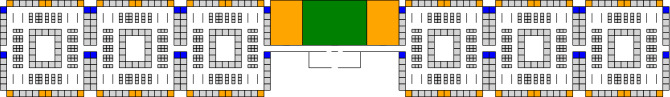
Floor plan representing the facility modeled in this work.

We use the facility layout shown in Fig. 3 representative of an office setting. The floor plan consists of a total of 828 office spaces (shown in grey), 48 meeting rooms (shown in orange, including two large ones), 1 cafeteria (shown in green), and 24 restrooms (shown in blue). The building has one large entrance area with hallway access to all the sections of the facility.

We vary the number of agents to achieve occupancy rates of 25%, 50%, 75% and 100%. The assignment of primary office locations for each agent is random but remains unchanged for all additional simulation runs at the specific occupancy level to ensure results can be aggregated and compared. The scheduling and meeting parameters used are given in Table 2 of the supplemental information and are representative of a typical office operation. We assume that 1 week of operation is enough to compute the daily probability of any two agents making contact (or in other words, weekly schedules don’t vary much from week to week) and run five 1-day ABM simulations at each occupancy rate to compute the contact probability matrix.

### Close contacts distribution and long-term transmission

**Figure 4:**
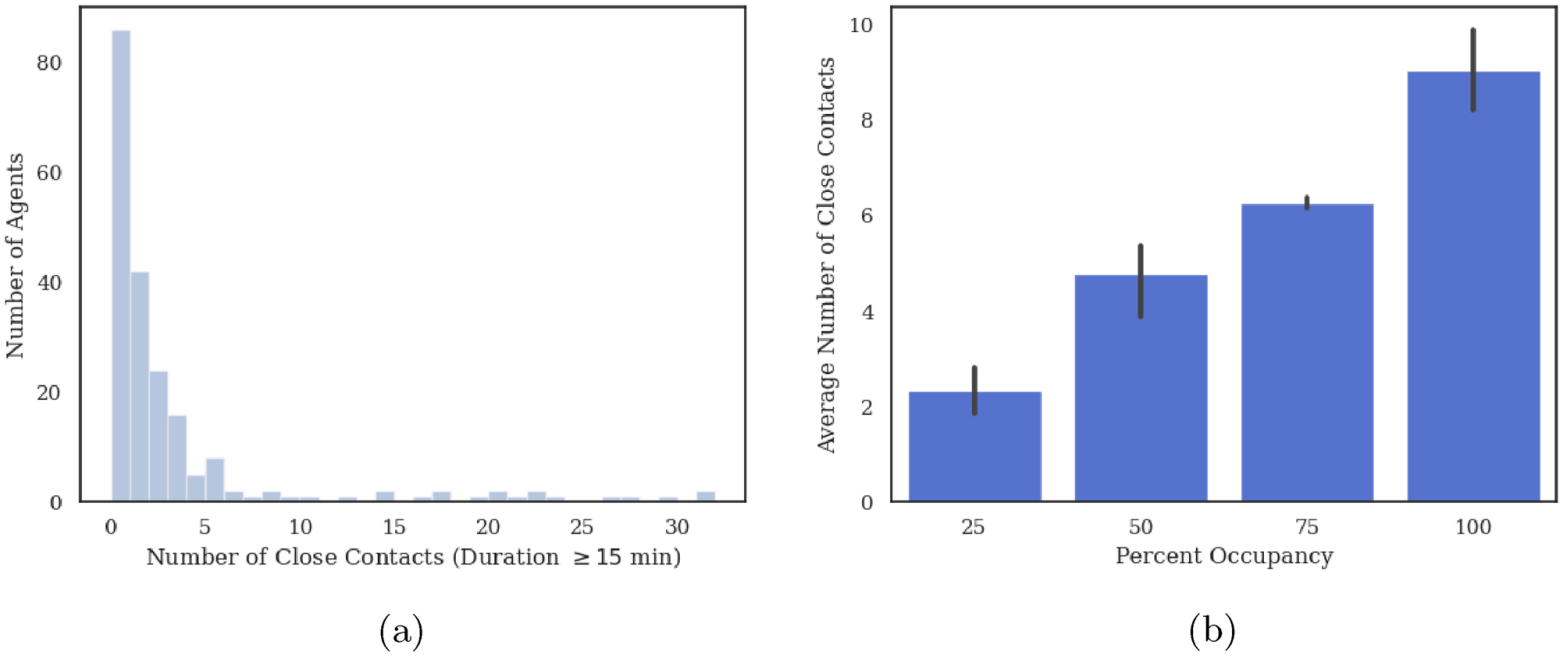
(**a**) Histogram showing the distribution of close contacts at 25% occupancy. The majority of agents have zero or a small number of close contacts while a few agents have a lot of close contacts. This trend is the same at all occupancy levels. (**b**) Average number of close contacts per agent as a function of facility occupancy level. As expected, the number of close contacts per agents increases as a function of facility occupancy underlying the importance of keeping occupancy as small as possible at any given time.

For each agent, we count the number of occurrences of a valid close contact event (cumulative contact duration greater or equal to 15 min) during a 9-h workday. In Fig. 4a we plot the resulting distribution of close contacts per agent under the scenario of 25% occupancy rate. The majority of agents (almost half of the population) have no close contacts at all and the vast majority of agents have less than 5 close contacts per day. However, a few agents have a relatively large number of close contacts ($\ge 10$). These agents are potential “super-spreaders”. If infected and asymptomatic, they will significantly drive the transmission rate within the facility. Figure 4b shows the average number of close contacts per agent, $1/2n\sum _{i}\sum _{j\ne i} f^{CDC}(D_{ij})$ where *n* is the number of agents which increases with the occupancy level in this facility going from 2.3 at 25% to 8.5 at 100%.

We use Eq. 2 to compute the probability of close contact using results from the parallel ABM simulations at each occupancy level. We then use the MC-LTTA to study indoor transmission over a 60-day time horizon. We perform 10 independent MC-LTTA runs for error estimation.

**Figure 5:**
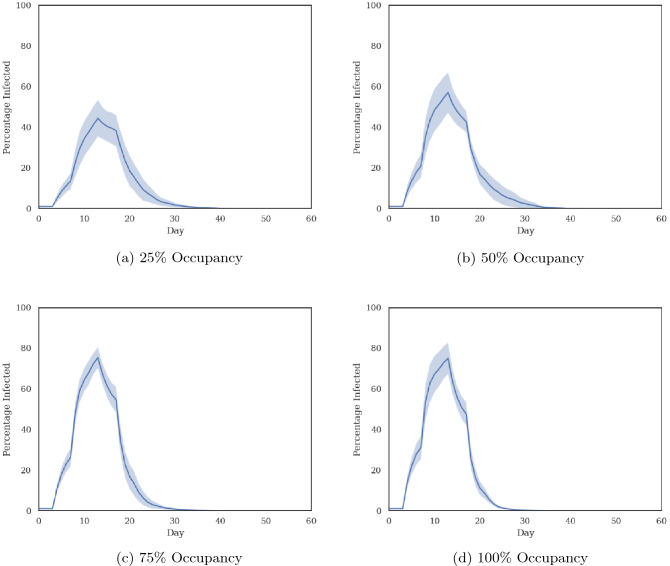
Prediction of the spread of the virus within the facility at different occupancy levels after 1% of the population is infected and asymptomatic.

Each MC-LTTA run starts with a total of 1% of facility users being infected and the remaining 99% being susceptible. We further assume that 40% of new infected individuals are asymptomatic^46^. Figure 5 shows the average and standard deviation of the percentage of infected individuals in the facility as a function of time, as computed from the MC-LTTA results at each occupancy level.

The number of infected individuals grows exponentially until the peak is reached and then decreases to zero as people recover from their infection. The relative number of individuals infected at the peak increases with occupancy level going from 45% to 80% respectively for 25% and 100% occupancy. This further illustrates the negative compounding effect of higher occupancy level which puts a higher percentage of facility users at risk.

Compared to larger scale infection studies (e.g., cities or countries) where all pairs of agents are assumed to have the same probability of close contact, two key differences from our simulation results are noticeable: (1) the timing of the peak is relatively constant across all occupancy levels and (2) the infection rate decays exponentially past the peak. Both of these effects can be attributed to the observation that some pairs of agents are more likely to have close contacts than others. Before the peak, most pairs of agents with non-zero probability of contacts actually make contact leading to a rapid spread of the infection among them. After the peak however, new infections hardly occur because people in the susceptible population who are still not infected are likely the ones with low probability of contact with other agents. In addition, currently infected individuals will reach their recovery period at the same rate as they were previously infected, leading to the observed trend.

### Network analysis of indoor outbreaks

**Figure 6:**
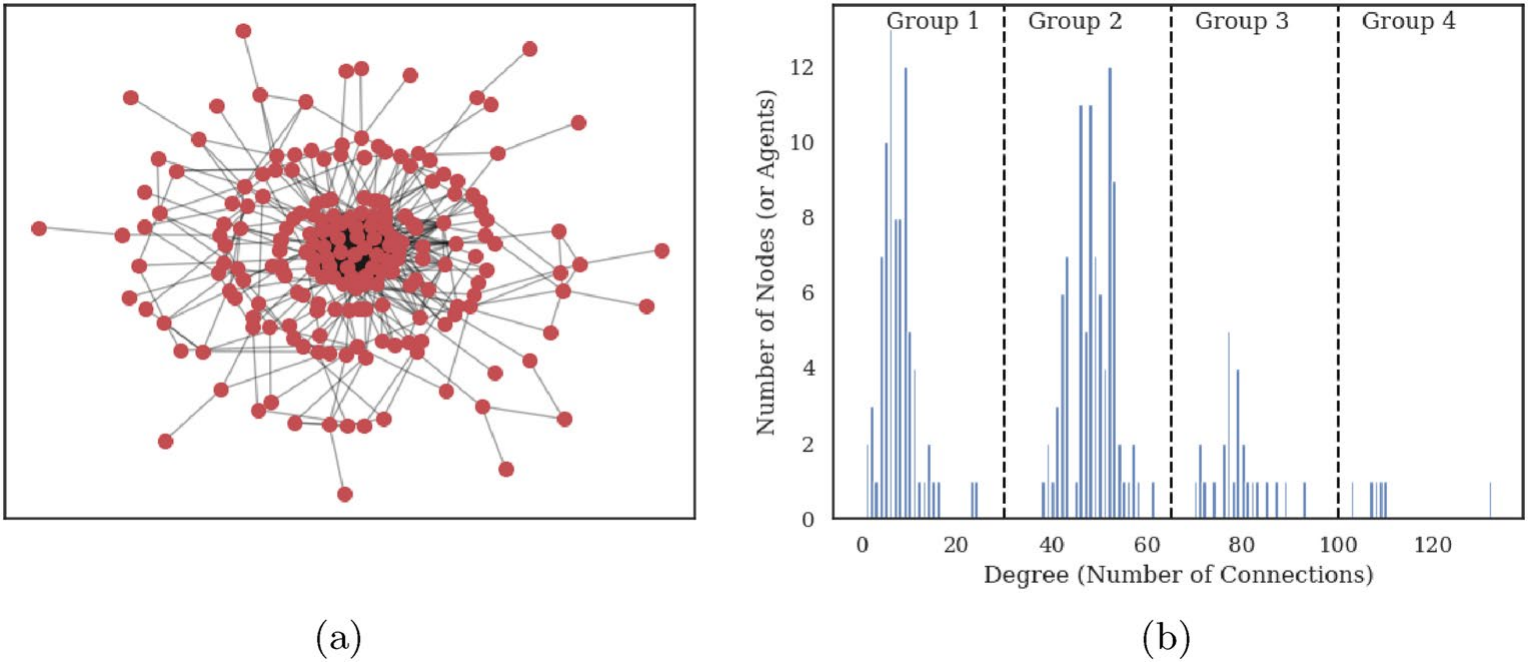
(**a**) Network analysis of the agents in the facility at 25% occupancy. Nodes represent agents and an edge exists between two agents if their probability of making contact is greater than zero. (**b**) Histogram of the degree (number of connections) of all the nodes in the network. Four groups of agents emerge naturally in terms of how connected they are to other agents.

In this section, we visualize facility users as a network in which a node represents an agent and an edge with other nodes is defined by their probability of close contact. Figure 6a shows such network for one of the simulations at 25% occupancy level. With the heterogeneous degeree (number of connections) distribution across all nodes, not all agents present the same risk of infection. Agents at the center are highly connected and therefore present a higher risk to start an outbreak in the facility if they are infected and asymptomatic. Such risk decreases significantly for agents further away from the center with far less connections. Similar results have been reported before where less than 20% of cases are responsible for more than 80% of transmissions^51^.

Within this framework, we can use the degree of each node to represent the likelihood to have close contacts with other agents. In Fig. 6b we show the degree histogram for all agents at 25% occupancy. The data indicate that several subgroups exist based on the number of connections of each agent: the first subgroup is made of agents with less than 30 connections; the second is with agents with 30 to 65 connections followed by 65 to 100 connections and finally more than 100 connections.

**Figure 7:**
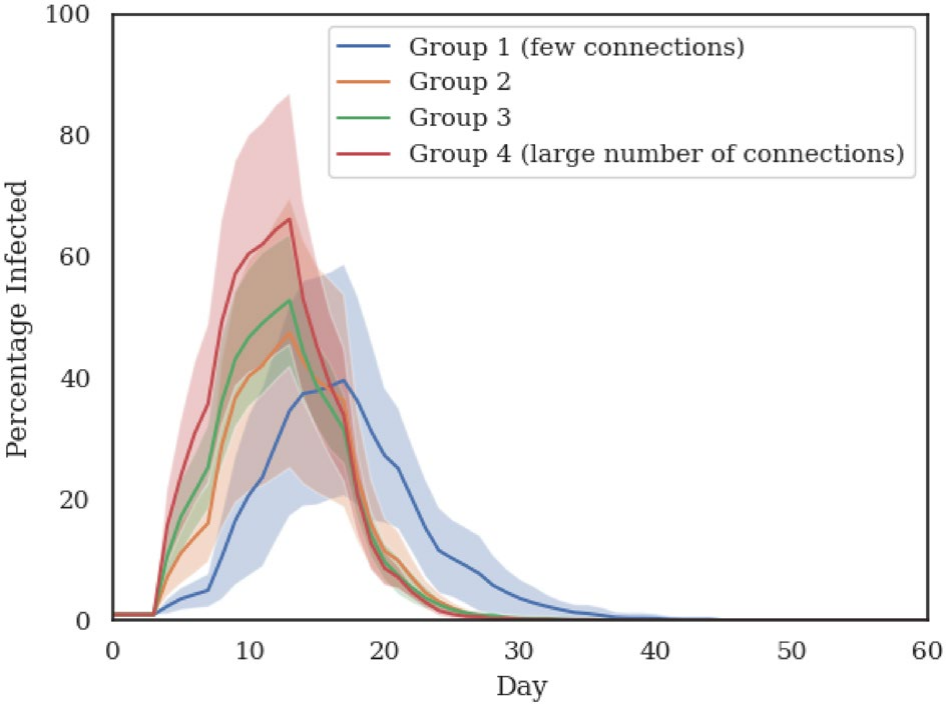
Spread of the virus at 25% occupancy, assuming all agents infected at outbreak onset are from a specific subgroup. The trajectory of the virus spread changes drastically based on who gets infected first. Agents with a lot of connections (group 4) are super-spreaders who can significantly alter the progression of the infection for the worse.

In Fig. 7 we plot four different outbreak profiles at the 25% occupancy level. In each case, the initial infected agents are restricted to a specific subgroup out of the four identified. These outbreak profiles differ from each other in two significant ways: (1) the position of the peak moves to the right as the initial infected agents are less connected (from subgroup 4 to 1) and therefore limited in the number of other people they can infect (2) the peak value increases as the “connectedness” of the initial infected agents increases. This means that not only more people get infected when the initial subgroup of asymptomatic agents routinely have close contacts with other agents but also the rate of disease infection spread is faster.

We also note that the decrease in infection after the peak is swifter with higher level of connectedness (i.e., it’s more abrupt for subgroup 4 compared to subgroup 1). This effect, as explained earlier, is amplified here because “slower outbreak trajectories” that would be created by agents with less connections are excluded by virtue of limiting the agents infected at outbreak onset to a specific subgroup.

Overall, these results show that all agents can potentially start an outbreak but not all outbreaks are equal. Highly connected individuals are likely to become super-spreaders if they are asymptomatic carriers. In the simulations discussed here, it increases the likelihood of starting a widespread outbreak by at least 4 times (in terms of peak infection rate) compared to individuals with low number of connections (or daily close contacts).

### Effect of facility layout

**Figure 8:**
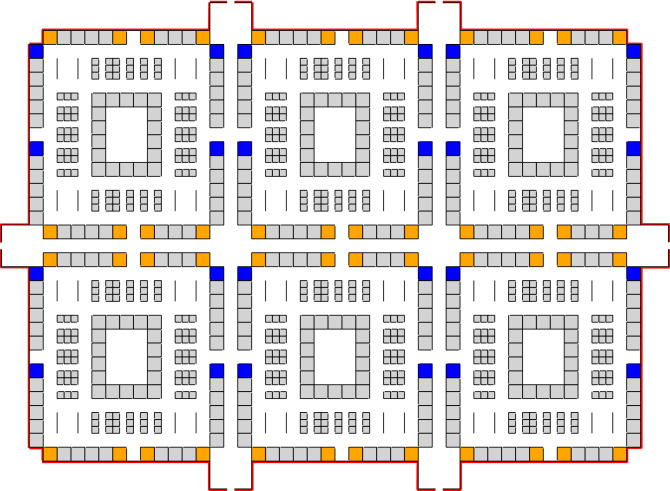
Modified layout of the facility with more entrance/exit doors, a more compact positioning of the various sections of the building and no cafeteria nor large meeting rooms. The total number of office spaces is relatively the same compared to the initial layout.

To explore the influence of facility layouts on the risk of infection, we introduce the following changes in the layout presented previously: (1) the relative position of each section is changed for a more compact arrangement and the number of entrances is increased from 1 to 6 allowing individuals to enter the facility closer to their assigned work space (2) auditorium and dining areas are removed altogether such that any large number of individuals can no longer congregate in central locations. The modified facility layout, with approximately the same capacity as the previous facility, is shown in Fig. 8.

**Figure 9:**
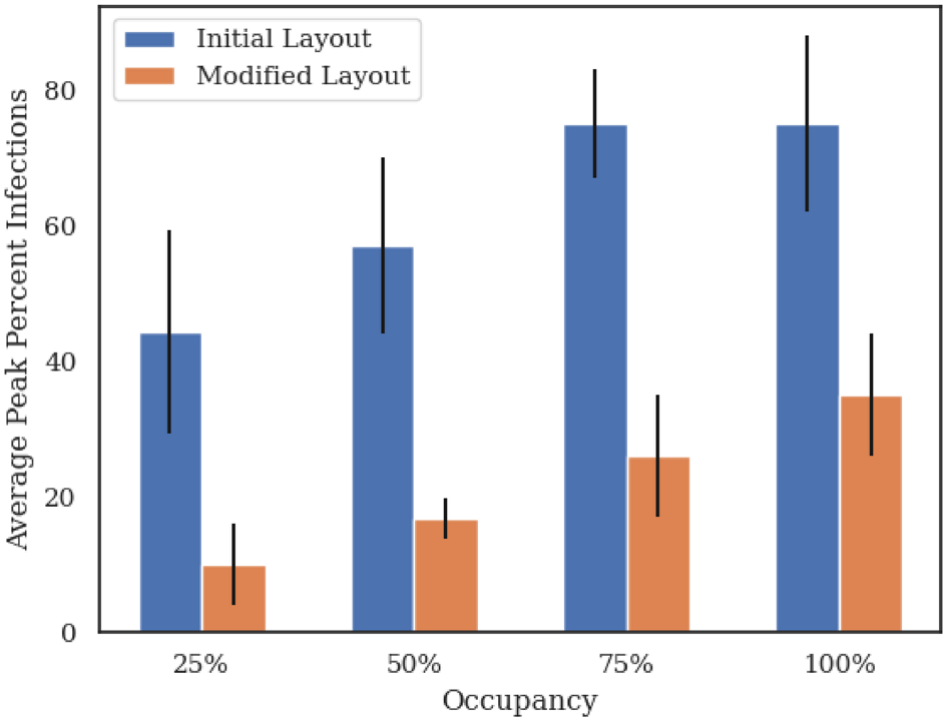
Comparison between average peak infections across two facilities with the same number of office spaces but different floor layouts at various occupancy rates. The modified layout with more entrance/exit doors and no large congregations exhibit 3-4x less infections illustrating how facility layouts and usage patterns play an important role in how indoor outbreaks start and evolve.

Figure 9 compares the average peak infection rate in both the initial and modified facility layouts across all occupancy levels. The modified layout shows a decrease in average peak infection rate three to four times that of the initial layout. By having multiple entrance doors allowing facility users to enter closer to their assigned workspace and eliminating any possibility for large indoor gatherings in meeting rooms or dining areas, we essentially divide the population into smaller sub-populations with little intermixing. This significantly reduces the probability of close contacts as well as the total number of people an individual can get into contact with, therefore leading to the observed decrease in peak infection rates.

## Discussion

### Identification of potential superspreaders

Overall, our results show that, in the scale of a single facility with long-term occupants, some individuals present a higher risk of spreading the virus if they get infected and are asymptomatic compared to other facility users. Therefore, ideally, special care should be taken to (1) identify potential super-spreaders and reduce their number of close contacts as soon as possible (2) ensure individuals identified as potential super-spreaders are adequately protected and closely monitored, preferably through testing even if they seem otherwise healthy^52^. Potential super-spreaders can be identified through a combination of mock contact tracing and network analysis to assign risk factors based on individual’s schedule, job function, and the facility where they work. The framework presented here can also be used to identify potential super-spreaders if each agent represent a real facility user. However, such a mitigation policy might not be practical as singling out particular individuals could imply discrimination and therefore is undesirable.

### Simulation of what if scenarios

Besides limiting the risk posed by potential super-spreaders, mitigation efforts will also benefit from simulating what-if scenarios to evaluate the efficacy of mitigation efforts and testing strategies before implementation. Such simulations should be performed separately for each facility of interest to capture the effects of floor layouts and behavior of occupants in that facility. They can help elucidate the relative influence of number of occupants, scheduling and meeting policies, number of entrances and exits, rate of transmission in the community surrounding the facility, rate of immunization, etc.

### Validation of simulation results

The proposed ABM modeling platform, which incorporates more critical and realistic perspectives of disease transmission than general statistical or mechanical models, can end up requiring more data to support its model parameterization need. Some data may be impossible to collect (i.e. transmission probability per contact) and others would demand a significant study preparation (i.e. schedule rules for staff in different functional areas). For the former, we recommend the users to apply advanced model calibration techniques such as approximate Bayesian computation^53^.

For the latter, we can provide some practical considerations specific to our modeling platform: i. Ensure the scheduling rules properly reflect the average behavior of facility occupants. Collect data on number of meetings, when people go to lunch, when they arrive and leave the facility, etc. ii. Ensure the ABM simulation duration matches the scheduling cycle of the facility (e.g. is the staff rotated every 4 days?) iii. Ensure the facility details are accurate after ingestion by the code iv. Check that the individual schedules generated by the code are realistic v. Verify that the traffic patterns automatically generated also match real traffic.

After the model parameterization and calibration processes are completed, the general rule of thumb is to compare the modeling outputs, which were not used to inform the calibration process, with the real data. For our model, one could compare the contact network structures resulting from the agents’ movement in the model with those recorded in some wearable devices by employees in the study to verify if the behavioral specifications are correct.

### Limitations

In this work, we focus on facilities that have predefined list of occupants such as office buildings, manufacturing facilities and schools. Other types of facilities with constantly changing occupants such as transit stations, restaurants, grocery stores, and event spaces (e.g., stadiums) were purposefully left outside of the scope of this work. To apply our method to those types of facilities, a distinction must be made between permanent (e.g., employees) and temporary occupants (e.g., customers). Additionally, the infection status of the latter will not be tracked over long-term horizons. Daily customer-to-customer transmissions can still be recorded but the primary focus for long-term analysis would be on customer-to-employee and employee-to-employee transmissions. Adding this category of temporary occupants will understandably introduce more uncertainty in the simulation results but will nevertheless be realistic and useful for insights into the most effective way to protect all facility users.

Another limitation of this work is that some behavioral choices that agents can make to avoid contacts are not considered. For example, agents may wear their mask, decide to stay as far apart as possible during a meeting, bring their lunch to work to avoid dining areas, etc. These factors can be added to the simulation framework by assigning average behavior expectations to agents to give them some agency in when and where contacts are made. However, from a mitigation strategy perspective, it’s probably better to make the more conservative choice of designing policies that can still work even if people make the wrong choices at an individual level.

## Conclusions

In this work, we introduce a new framework to assess the risks of outbreaks in indoor environments. The proposed framework supports real floor plans and relies on ABM simulations to compute probability of close-contacts between facility users coupled with a Metropolis Monte Carlo algorithm for long-term transmission studies. It enables realistic contact networks to be generated within a specific facility without relying on wearable devices or intrusive camera surveillance. It also enables facility administrators to explore the tradeoffs between various policy options to control the spread of infectious diseases. We demonstrate the use of this framework on two different facility layouts at various occupancy levels. Our results confirm that limiting the number of facility users lowers the risk of outbreak, and that facility layouts and scheduling rules that can partition users into groups with little intermixing can contribute to lowering the risk of outbreaks. By doing a network analysis on facility users, we also show that some facility users present a higher risk of starting an outbreak and discuss related mitigation strategies. With the open-source release of our customizable ABM platform that supports floor plans of real facilities, we hope this work will be helpful in combating novel infectuous diseases, including COVID-19, in indoor environments.

## Supplementary Information

Supplementary Information.

## Data Availability

The agent-based modeling code is open-source and available to the public in this GitHub repository: https://github.com/corning-incorporated/citam.
