# Supplementary material for: Predicting and preventing COVID-19 outbreaks in indoor environments: an agent-based modeling study
Source: Sci Rep. 2022 Sep 27;12:16076. doi: 10.1038/s41598-022-18284-8 (PMC9514194; doi:10.1038/s41598-022-18284-8)
Supplement: Supplementary file 1 — Supplementary Information. [file 41598_2022_18284_MOESM1_ESM.pdf]

Predicting and Preventing COVID-19 Outbreaks in Indoor Environments: An Agent-Based Modeling Study

Mardochee Reveil*[1] and Yao-Hsuan Chen[2]

[1]Corning Research and Development Corporation
[2]Merck & Co., Inc., Kenilworth, NJ, USA

May 16, 2022

1 Agent-Based Modeling Scheduling Parameters

Table 1 shows the parameter values used to create daily schedules for each agent in the facility. The first column is the type of activity that the agent does. The location of the activity is randomly chosen from a list of compatible locations in the facility. The duration of the activity is randomly chosen between the minimum and maximum duration. The number of occurrences during the day is also randomly chosen between the minimum and maximum number of occurrences. All random sampling for scheduling purposes assume a uniform distribution.

Name	Minimum Duration	Maximum Duration	Minimum Occurrences	Maximum Occurrences
Office Work	10 min	120 min	2	10
Restroom Visit	5 min	15 min	1	4
Cafeteria Visit	15 min	60 min	0	2
Meeting	20 min	120 min	1	8

Table 1: Scheduling parameters used in the ABM simulations.

2 Monte-Carlo Long-Term Transmission Inputs

2.1 Algorithm

A pseudo-code description of the Monte-Carlo Long-Term Transmission Algorithm is provided here as a support for implementation.

Algorithm 1 Monte Carlo Long-Term Transmission Algorithm

```
Require: asymp_prob, contact_prob
for day ← 1 to n_days do
    new_asymp ← []
    new_symp ← []
    for all infectuous_agent do
        for all susceptible_agent do
            pc ← contact_prob[asymptomatic_agent][susceptible_agent]
            if random() ≤ pc then
                if random() ≤ asym_prob then
                    new_asymp.append(agent)
                else
                    new_symp.append(agent)
                end if
            end if
        end for
        update_infected_agents(new_asymp, new_symp)
        update_non_susc_agents()
    end for
end for
```

2.2 MC-LTTA Inputs

Table 2 shows the list of parameters required for the Monte-Carlo Long-Term Transmission Algorithm (MC-LTTA). Some of those parameters are based on the epidemiology of the disease of interest and are, essentially, the probability for an infected individual to be asymptomatic for the duration of the infection, the first day after infection when an agent becomes contagious and the last day of their contagious period. In this study, we use the values of 0.4, 3 and 14 respectively for these parameters based on relevant reports about COVID-19 in the literature. A sensitivity analysis around these values is presented in the following section. The remaining parameters are user inputs such as the number of agents, the number of days to run the simulation for and the initial list of asymptomatic (or infected) individuals. As mentioned before, the pair contact probability computed from the ABM simulations is also a key input.

Name	Type	Description
n_agents	integer	Number of agents
asymp_agents	array of arrays	List of infected asymptomatic agents grouped by day after infection.
non_susc_agents	array	List of agents that cannot be infected whether because they have already recovered or have been immunized.
n_days	integer	Number of days to run the simulation for
contact_prob	symmetric matrix	Pair probability of close contact
asymp_prob	float	Probability that an infected agent is asymptomatic
presymp_period	integer	Days between when an agent becomes contagious and when they start developing symptoms
first_cont_day	integer	First day after infection when agent becomes contagious
last_cont_day	integer	Day after infection when agent is no longer contagious

Table 2: Parameters used in the Monte-Carlo Long-Term Transmission algorithm described in this work.

3 Sensitivity Analysis

We explore the influence of the following parameters: the latency of the disease (how long it takes before infected individuals become contagious), the percentage of the population that's infected at the beginning of the simulation, the probability that an infected individual is asymptomatic, and the duration of any pre-symptomatic period. In all cases, we repeat the simulations for the first facility described in the paper and at half occupancy.

3.1 Latency

Figure 1 shows the influence of the latency value on the percentage of infections as a function of time. We use latency values of 1 to 4 days. As expected, the population of infected individuals starts increasing only after the initial latency period is completed. We also observe that the curves for lower latency value are smoother than the ones at higher latency values. This because at higher latency values, the transmission dynamic is similar to a cohort-based transmission whereby a group of individuals will get infected, go through the disease progression process and recover together.

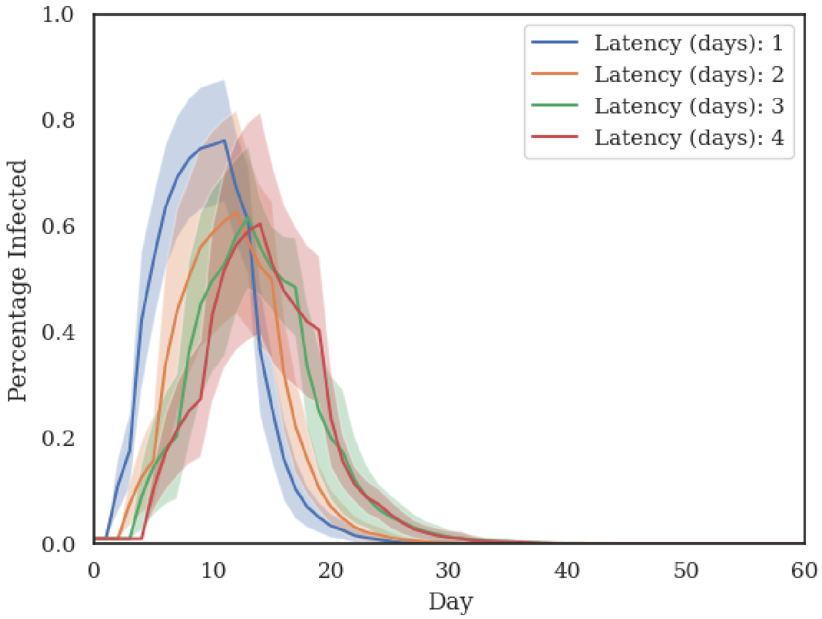

Figure 1: Effect of the latency or incubation period.

3.2 Initial Infections

Figure 2 shows how the number of initial infections influences the progression of the disease within the facility. We use initial infection values of 0.5, 1.0, 2.0 and 4.0%. As expected, the higher the initial number of infections, the faster the disease is transmitted within the facility and the higher the peak infections unless other preventative measures are taken. The results presented in the paper are for an initial percentage of infections of 1.0%.

3.3 Asymptomatic Probability

In Fig. **??** we vary the asymptomatic probability from 25% to 40%. The results show that the trajectory of the disease within the facility does not change significantly as a function of the asymptomatic probability in the 25% to 40% regime. We use a value of 40 % in the paper.

3.4 Presymptomatic Period

Figure 4 shows the influence of the duration of the presymptomatic period varying from 1 to 3 days. The results indicate that the exact value of the presymptomatic period does not significantly influence the disease progression

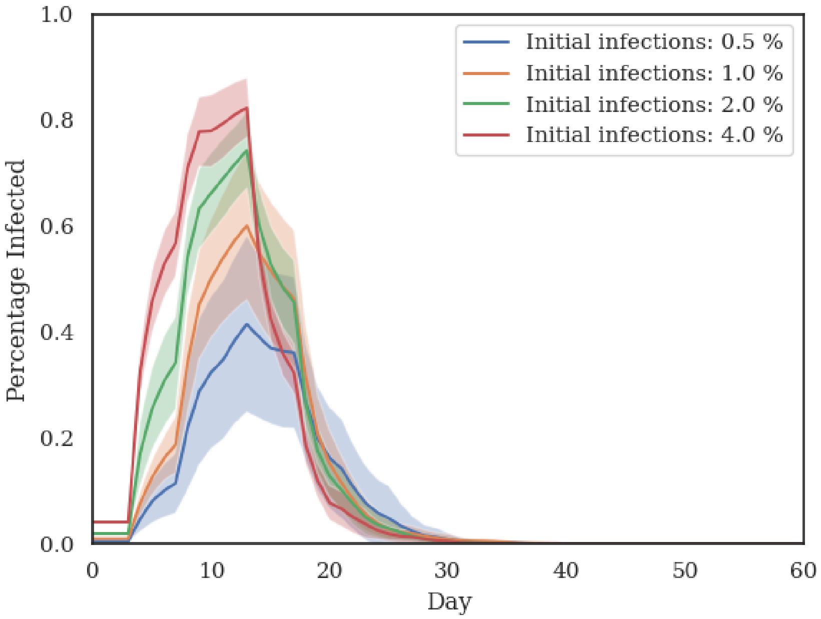

Figure 2: Effect of the number of initial infections.

within the facility. Having people who don't show any symptoms but can still infect other people is always detrimental.

4 Considerations for Implementation

In this work, we use the CDC definition of close contact to model the transmission risk as a delta function using a proximity distance and cumulative duration cutoffs of 6 ft and 15 min respectively. As mentioned before, a delta function is not the most realistic way to model the transmission probability for at least two reasons: (1) transmission can occur when individuals are more than 6 ft apart and have been in contact for less than 15 min and (2) it doesn't take into account other important factors that may drive the transmission risk. Several publications have sought to derive expressions for the highly complex and multivariate transmission risk of a respiratory infection such as COVID-19 by taking into account respiratory droplet [1, 2], sneezing and coughing dynamics [3, 4], a comprehensive list of factors [5], etc. One or more of these expressions can be used within our proposed framework to obtain results that are more realistic. Similarly, as more data is gathered about new strains of SARS-COV-2, the transmission function can be updated accordingly.

Although the full importance of airborne transmission has not been estab-

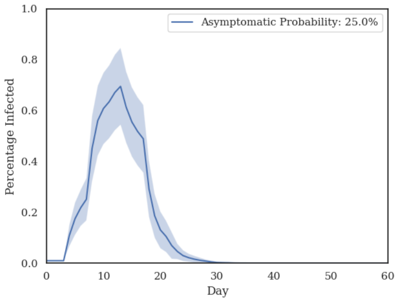

(a) 25% asymptomatic probability

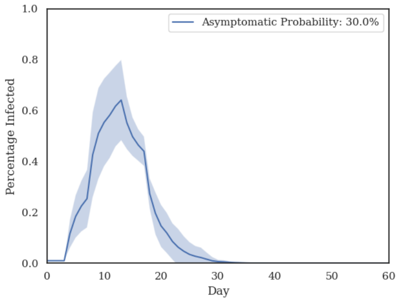

(b) 30% asymptomatic probability

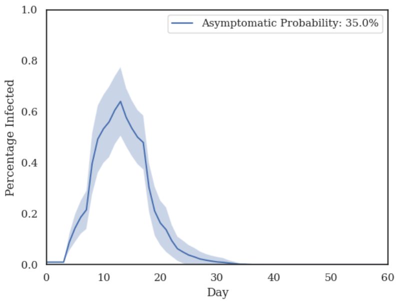

(c) 35% asymptomatic probability

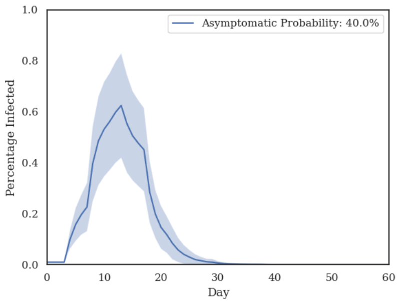

(d) 40% asymptomatic probability

Figure 3: Effect of the asymptomatic probability value.

lished yet, several studies have suggested that stagnant air with small droplet nuclei that includes the virus [6] as well as contaminated surfaces are potential infection routes. In other words, there is a non-zero probability that users of a facility get exposed to the virus without ever having direct contact with infected individuals. While this risk factor is left outside of the scope of this work, indoor air quality (e.g. quality of the ventilation, humidity, temperature, etc.) and surface cleaning policies can be used to assign a probability of agents getting infected without direct contact. This probability of infection can be incorporated into the MC-LTTA framework in a similar way that outside infections are incorporated.

References

[1] S. Balachandar, S. Zaleski, A. Soldati, G. Ahmadi, and L. Bourouiba. Host-to-host airborne transmission as a multiphase flow problem for science-based social distance guidelines. *International Journal of Multiphase Flow*, 132:103439, 2020.

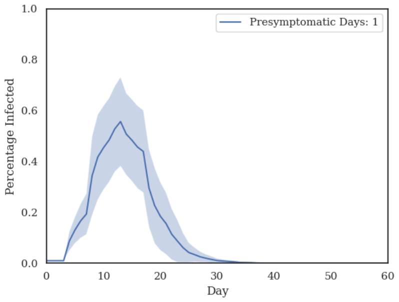

(a) 1 day

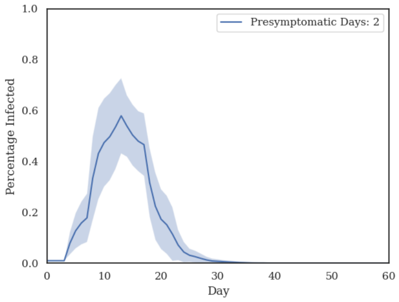

(b) 2 days

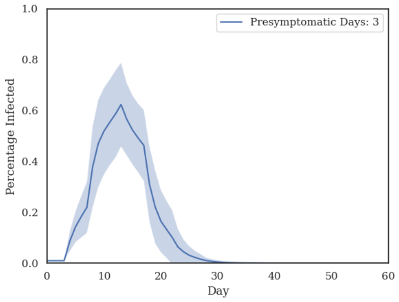

(c) 3 days

Figure 4: Effect of the presymptomatic period.

[2] Swetaprovo Chaudhuri, Saptarshi Basu, and Abhishek Saha. Analyzing the dominant sars-cov-2 transmission routes toward an ab initio disease spread model. *Physics of fluids (Woodbury, N.Y. : 1994)*, 32(12):123306–123306, Dec 2020. 33311972[pmid].

[3] Giacomo Busco, Se Ro Yang, Joseph Seo, and Yassin A. Hassan. Sneezing and asymptomatic virus transmission. *Physics of fluids (Woodbury, N.Y. : 1994)*, 32(7):073309–073309, Jul 2020. 32684746[pmid].

[4] Talib Dbouk and Dimitris Drikakis. On coughing and airborne droplet transmission to humans. *Physics of fluids (Woodbury, N.Y. : 1994)*, 32(5):053310–053310, May 2020. 32574229[pmid].

[5] Rajat Mittal, Charles Meneveau, and Wen Wu. A mathematical framework for estimating risk of airborne transmission of covid-19 with application to face mask use and social distancing. *Physics of fluids (Woodbury, N.Y. : 1994)*, 32(10):101903–101903, Oct 2020. 33100806[pmid].

[6] G. Aernout Somsen, Cees van Rijn, Stefan Kooij, Reinout A. Bem, and Daniel Bonn. Small droplet aerosols in poorly ventilated spaces and sars-

cov-2 transmission. *The Lancet. Respiratory medicine*, 8(7):658–659, Jul 2020. 32473123[pmid].
